# Reconstructive and Oncoplastic Surgery for Giant Phyllodes Tumors: A Single Center’s Experience

**Published:** 2017-05

**Authors:** Vassilis Pitsinis, Osama Moussa, Fiona Hogg, Jane McCaskill

**Affiliations:** Breast Unit of Tayside NHS Trust, Ninewells Hospital and Medical School, Dundee, Scotland

**Keywords:** Reconstructive surgery, Oncoplastic surgery, Giant phyllodes tumors

## Abstract

Phyllodes tumors are biphasic fibroepithelial neoplasms of the breast. While the surgical management of these relatively uncommon tumors has been addressed in the literature, few reports have commented on the surgical approach to tumors greater than ten centimetres in diameter – the giant phyllodes tumor. We report a case of giant breast tumors and discuss the techniques utilized for pre-operative diagnosis, tumor removal, and breast reconstruction. A review of the literature on the surgical management of phyllodes tumors was performed.

Management of the large phyllodes tumors presents the surgeon with unique challenges. The majority of these tumors can be managed by simple mastectomy but reconstruction and even oncoplastic conservative management is for selective consideration.

## INTRODUCTION

Cystosarcoma phyllodes is a rare, predominantly benign of unknown aetiology breast tumor that occurs almost exclusively in the female. Its name is derived from the Greek words sarcoma (“fleshy tumor”), and phyllon (“leaf”). Grossly the tumor displays characteristics of a large, malignant sarcoma, and takes on a leaf like appearance when sectioned histologically. Because most tumors are benign, the name may be misleading. Thus, the favoured terminology is now phyllodes tumor (PT). Phyllodes tumors accounts for 0.5% of all breast tumors. Mean age of diagnosis is 35-40 years and the 80% of patients are in the premenopausal period of their lives.^1^ Until the late 1970s mastectomy was the standard surgical treatment for all phyllodes irrespective of tumor size or histological type.^2^ Radical surgery compared offers no survival advantage and many surgical options are now available.^3^

## CASE REPORT

A 56-year-old woman presented to us with a 5-month history of rapidly growing very large left breast swelling. She self-presented mainly due to excessive bleeding and foul smelling excoriation of the skin. On initial assessment the patient had no comorbidities, no hormonal background though a positive family history of breast carcinoma. Clinical examination revealed a generalised obvious mass of the left breast. The skin of the breast was enlarged and excoriated with a large necrotic foul-smelling antero-lateral aspect. There was no palpable adenopathy in either of the axilla.

**Fig. 1:**
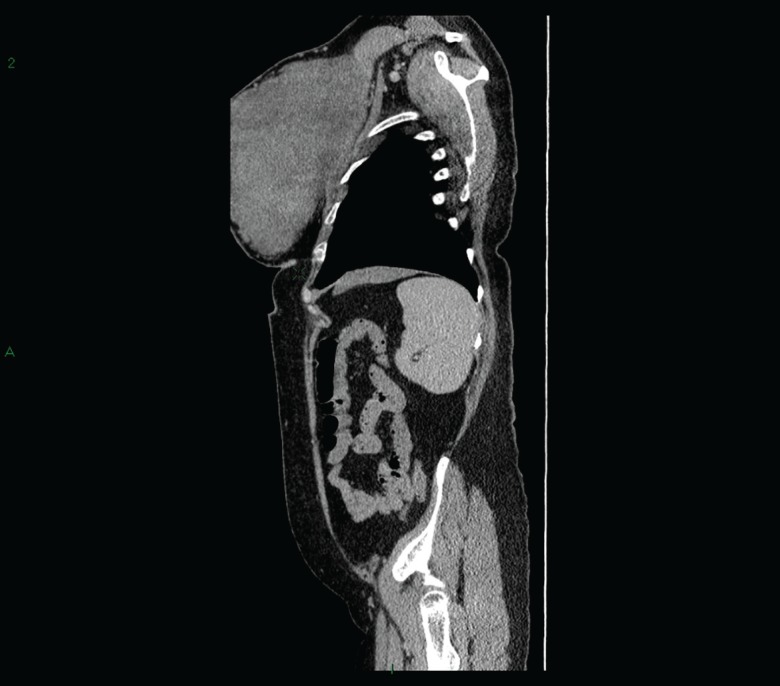
CT revealed a large solid heterogeneous left breast mass going off the CT window measuring at least 20 cm in a single oblique measurement

Initial triple assessment involved ultrasound which suggested a gigantic vascular tumor which exhibited Doppler ﬂow and proceeded to an Ultrasound guided core biopsy after informed consent, histology revealed a phyllodes tumor. It was decided to proceed to a CT chest, abdomen and pelvis and subsequently to plan a mastectomy to consider immediate reconstruction. CT revealed a large solid heterogeneous left breast mass going off the CT window measuring at least 20 cm in a single oblique measurement (Figure 1). No focal regions of necrosis could be identified. Borderline left pectoral lymph nodes the largest measuring up to 10 mm. Subcentimetre nodes are identified within the right axilla. No evidence of any focal pulmonary metastases. No evidence of direct rib invasion from the large left breast mass. 

**Fig. 2A, B:**
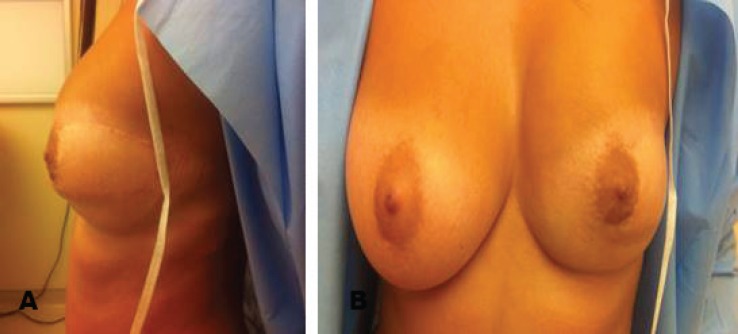
The challenging mastectomy case due to the peri-tumoral inflammation and increased vascularity

The patient underwent mastectomy and an immediate deep inferior epigastric perforator reconstruction. The mastectomy was challenging due to the peri-tumoral inflammation and increased vascularity (Figure 2A). Post-operatively early venous congestion necessitated re-operation; however medial flap necrosis was not avoided. Definitive histology showed a malignant Phyllodes tumor. Microscopy showed a biphasic tumor with a spindle cell stroma and epithelial elements. The features were in keeping with a phyllodes tumor in view of the cellularity mitotic rate and necrosis and hence classified as a malignant phyllodes tumor.

Similar pathological characteristics had the second case of a 27 year old lady with previous history of fibroadenoma excision that presented with a 14cm upper outer quadrant tumor attached the overlying skin. Ultrasound guided core biopsy revealed a phyllodes tumor. The patient had a normal clinical and ultrasonographicaly normal axilla thus no axillary dissection was pursued. In view of the need of skin excision to achieve margins we opted for the use of a nipple sparing mastectomy with immediate latissimus dorsi muscle musculocutaneous flap and implant reconstruction. 

The incision was extended laterally to involve the skin of the upper outer quadrant which was excised en block with the breast and which was replaced by the island of skin from the latissimus musculocuateous flap (Figure 2B). In addition the parts pectoral and serratus muscles deep to the phylloides tumor were also excised to achieve adequate macroscopic margins. Microscopy showed high stromal cellularity, leaf-like clefting, variable stromal overgrowth, focal cellular atypia and mitotic activity (up to 5 per 10 hpf) and invasive edges. The lesion was best classified as phyllodes tumor with borderline malignant potential. 

**Fig. 3:**
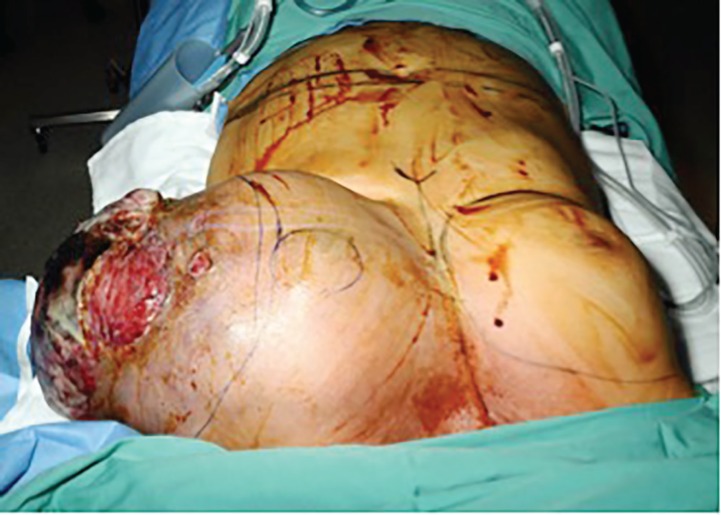
The incision extending laterally to involve the skin of the upper outer quadrant excised en block with the breast and replaced by the island of skin from the latissimus musculocuateous flap. At one point the lesion was seen invading into skeletal muscle. All excision margins were clear

At one point the lesion was seen invading into skeletal muscle. All excision margins were clear. Postoperative recovery was uneventful and besides some lack of volume in the upper quadrant the patient was satisfied with the overall cosmetic result (Figure 3). The patient did not receive any radiotherapy and remains disease free at 5 years of follow up. 

## DISCUSSION

The phyllodes tumor (PT), originally described by Johannes Muller in 1838, has presented a diagnostic and treatment dilemma for physicians since its original description. This tumor is structurally analogous to fibroadenoma but differs from it histologically by the presence of leaf like projections of stroma and increased stromal cellularity.^4^ Twenty percent of tumors grow larger than 10 cm, the arbitrary cut off point for the designation as a giant tumor.^5^ These tumors can reach sizes up to 40 cm in diameter.^6^

PT usually presents as a rapidly growing and clinically benign breast lump in females. Phyllodes tumors can grow to significant sizes especially if presentation is delayed. Given that wide excision is essential, mastectomy may be the required surgical option. For such cases reconstruction will be challenging due to hostile operative environment, which may well have contributed in our case to partial flap loss and therefore meticulous, collaborative pre-operative planning is essential to maximise the chances of a satisfactory oncoplastic outcome. Simple intracapsular enucleation “shelling out” irrespective of histological type results in an unacceptably high rate of local recurrence.^7^

In a large study of 165 patients from the Insitut Currie in Paris the surgical management of 3 cm median in size tumors was examined. The majority (97%) of patients received simple not wide local excision with only 3% having received oncoplastic wide local excision or mastectomy operations. The 28% of cases had infiltrated margins and the reoperation rate reached 52%.^8^ Most authors consider excision margin to be positive if the tumor was present at or close to (<1 mm) the inked tissue on fistopathology.^5^ In fact, taking into account that local recurrence of phyllodes is high and that an affected margin is the single independent predictor of local recurrence is therefore understandable the paramount significance of the wide excision in the local recurrence-free rate.^5^^,^^9^

Wide excision or mastectomy provided that surgical margins were adequate yielded similar high local control rates in similar grade PTs.^10^ The largest published review on the long-term outcomes in relation to clinicopathological features from the Memorial Sloan Kettering Cancer Centre looking at 293 Phyloides tumors over a period of nearly 60 years confirmed that local treatment failures occurred with equal frequency in benign and malignant tumors. In the same review is worth to mention the fact that local recurrence rate between conservation and mastectomy were not statistically significant provided the margins of excision were adequate.^5^ To strengthen the importance of adequate margins it is important to note that in a retrospective study of recurrent PTs from Singapore it was shown that tumor grade did not influence the adequacy of surgical margins or the time interval to local recurrence or number the of recurrences. Besides the fact that malignant tumors tended to recur earlier, this was also not found to be statistically significant.^11^

Two other factors associated with increased rates of local recurrence was fibroepithelial proliferation in the surrounding breast tissue, necrosis and a history of fibroadenoma. However with the exception of necrosis each of the above characteristics can be seen in benign PTs.^5^ Adjuvant radiotherapy was used in cases of higher-grade tumors in the past.^12^ Furthermore, an observational study reported a trend toward increased utilisation of radiotherapy with the rational of improved survival and reduced recurrence.^13^ The case of improved survival was not proven from the Analysis of the National Cancer Data Base, 1998–2009 in the USA concluding that it had no effect on disease free survival (DFS) or overall survival (OS).^14^

The recommendations of the MD Anderson Cancer centre are to consider radiotherapy for malignant PTs in the setting of local recurrence.^15^ In a recent systematic review and meta-analysis of the effects of radiotherapy on borderline and malignant phyloides tumors it was suggested that there is not an observed significant difference in overall survival or disease free survival for those receiving it.^16^ In addition, its role is unclear and has not been the subject of large randomised control trials.^17^ We hope that the outcomes of the radiation therapy after surgery in treating women with phyllodes tumor of the breast trial from the USA with an estimated completion date in December 2019 will elucidate us on this controversial issue.^18^

Although limited in the literature, reports of immediate breast reconstruction have been produced.^5^^,^^19^ Immediate breast reconstruction with implant only autologus tissue alone or in combination with an implant did not warrant a higher recurrence rate compared to simple mastectomy and also it did not interfere with follow up or the detection of recurrent lesions.^19^^-^^21^ In the appropriate setting nipple sparing skin-sparing mastectomy can be applied without affecting recurrence rates something that is in the author’s previous personal experience. The same applies for breast conservation oncoplastic approach in the treatment of wide excisions and local recurrence being no different provided adequate margins are achieved.^22^

Management of the large phyllodes tumors presents the surgeon with unique challenges. Complete excision, with accurate histologic examination and continued follow-up care, is the best way to treat phyllodes tumors. In most cases wide local excision is indicated, with a rim of normal tissue included. Although no absolute rules regarding margin size have been established a 1 cm macroscopic at the time of the operation and 1mm microscopic margin for most cases has been advocated as adequate.

The lesion should not be “shelled out,” as might be done with a fibroadenoma, or the recurrence rate will be unacceptably high. That makes preoperative histological diagnosis very important. If the tumor-to-breast ratio is sufficiently high to preclude a satisfactory cosmetic result with any oncoplastic excision, total mastectomy, with or without reconstruction, is an alternative. More radical procedures generally are not warranted. 

## CONFLICT OF INTEREST

The authors declare no conflict of interest.
